# Supplementary material for: Thiol-maleimide poly(ethylene glycol) crosslinking of L-asparaginase subunits at recombinant cysteine residues introduced by mutagenesis
Source: PLoS One. 2018 Jul 27;13(7):e0197643. doi: 10.1371/journal.pone.0197643 (PMC6063399; doi:10.1371/journal.pone.0197643)
Supplement: S1 File — (PDF) [file pone.0197643.s001.pdf]

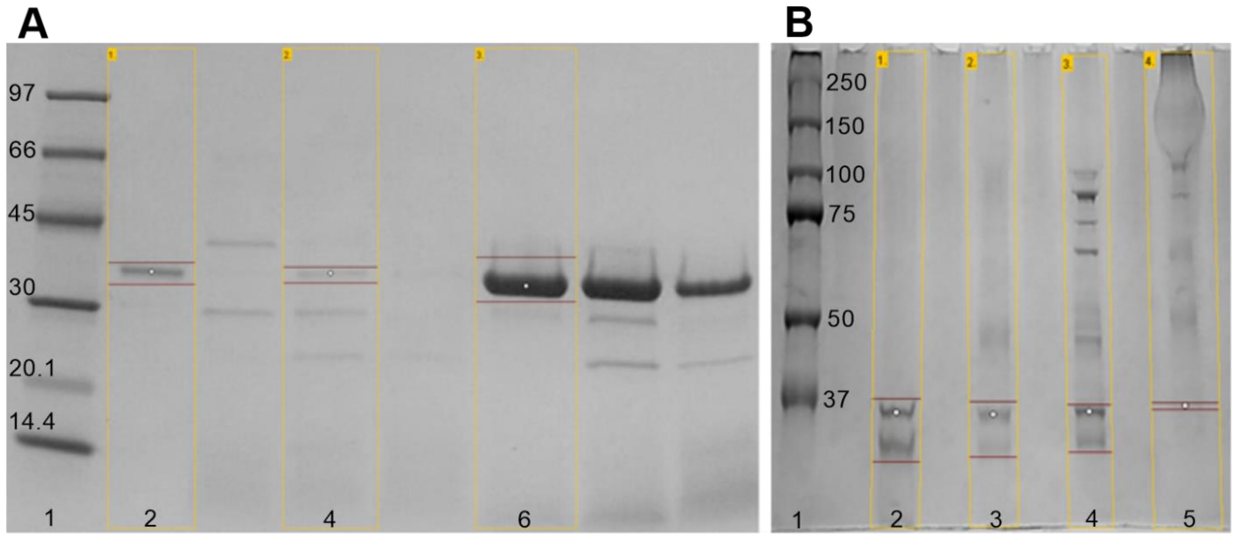

S1 Fig. Densitometry analysis of SDS-PAGE gels. The band intensities from SDS-PAGE gels were calculated by densitometry analysis to correct L-asparaginase concentration from the total protein values calculated by the BCA method. (A) Correction for the C77-105S mutant L-asparaginase. [1] Molecular weight marker, [2] commercial natural L-asparaginase (Millipore Sigma, USA), [4] C77-105S mutant, [6] recombinant native L-asparaginase. (B) Correction for the 5kDa-PEG-conjugate. [1] Molecular weight marker, [2] recombinant native L-asparaginase, [3] commercial randomly-PEGylated L-asparaginase (Millipore Sigma, USA), [4] non-conjugated A38C-T263C mutant, [5] 5kDa-PEG-conjugate. Bands for non-conjugated L-asparaginase are selected in red. SDS-PAGE electrophoresis (B) was run for 3 h to allow the 5kDa-PEG-conjugate to migrate. Note the low purity of the starting mutant solution in (B).
