# Supplementary material for: Thiol-maleimide poly(ethylene glycol) crosslinking of L-asparaginase subunits at recombinant cysteine residues introduced by mutagenesis
Source: PLoS One. 2018 Jul 27;13(7):e0197643. doi: 10.1371/journal.pone.0197643 (PMC6063399; doi:10.1371/journal.pone.0197643)
Supplement: S2 File — (PDF) [file pone.0197643.s002.pdf]

S2 Table. Recovery of cysteine-directed PEGylation crosslinking by ultrafiltration.

Step	Volumetric activity (U/ml)	Volume (ml)	Total activity (U)	Recovery (%)
Sephadex desalting	16.0	3.50	56.1	100
TCEP Reduction	112	0.50	56.2	100
5kDa-PEG-conjugate[a]	158	0.17	26.9	47.9
Non-conjugated[b]	41.0	0.60	24.6	43.8

[a]The 5kDa-PEG-conjugate was pooled from a 100 kDa cut-off filter.

[b]The flow-through from the 100 kDa cut-off filter corresponds to non-conjugated or small bio-PEG-conjugates, which was concentrated and pooled from a 10 kDa cut-off filter.
