# Supplementary material for: Thiol-maleimide poly(ethylene glycol) crosslinking of L-asparaginase subunits at recombinant cysteine residues introduced by mutagenesis
Source: PLoS One. 2018 Jul 27;13(7):e0197643. doi: 10.1371/journal.pone.0197643 (PMC6063399; doi:10.1371/journal.pone.0197643)
Supplement: S3 File — (PDF) [file pone.0197643.s003.pdf]

**S3 Table. Correction of L-asparaginase concentration derived from densitometry analysis.**

| Sample | **Total protein conc.** (mg/ml) | **Vol. loaded into gel** (µl) | **Band intensity** (IU) | **Mass loaded into gel** (µg) | **Asparaginase conc.** (mg/ml) |
|---|---|---|---|---|---|
| Native[a] | 0.136 | 9.40 | 1321 | 1.28 | **0.136** |
| A38C-T263C | 1.11 | 1.20 | 954 | 0.923 | **0.769** |
| 5kDa-PEG-conjugate | 0.622 | 2.20 | 11 | 0.0106 | **0.617** |
| Randomly-PEGylated | 0.171 | 7.60 | 619 | 0.599 | **0.0922** |
| Natural[b] | 0.200 | 10.00 | 404 | 2.00 | **0.200** |
| Native | 1.13 | 10.00 | 2682 | 13.3 | **1.33** |
| C77-105S | 0.0430 | 10.00 | 78 | 0.386 | **0.0386** |

For the 5kDa-PEG-conjugate and randomly-PEGylated L-asparaginases, the concentration from densitometry of non-conjugated L-asparaginase bands was subtracted from the total protein concentration obtained by the BCA method to yield the final L-asparaginase concentration.

[a,b]Commercial natural and recombinant native L-asparaginases were used as control to calculate the L-asparaginase concentration based on the direct ratio of band intensities.
