# Supplementary material for: Thiol-maleimide poly(ethylene glycol) crosslinking of L-asparaginase subunits at recombinant cysteine residues introduced by mutagenesis
Source: PLoS One. 2018 Jul 27;13(7):e0197643. doi: 10.1371/journal.pone.0197643 (PMC6063399; doi:10.1371/journal.pone.0197643)
Supplement: S4 File — (PDF) [file pone.0197643.s004.pdf]

S4 Table. Calculation of asparaginase specific catalytic activity.

Sample	$OD_{425nm\ blanked}$ (AU)			Volumetric activity[a] (U/ml)	Conc. dilution (*x*)	Conc. in Rxn (mg/ml)	Specific activity (U/mg)
Natural	0.974	1.029	1.026	7.65 ± 0.29	4.6	0.0432	177 ± 11
Native	1.121	1.001	1.022	7.94 ± 0.60	26	0.0508	156 ± 14
C77-105S	0.813	0.805	0.833	6.19 ± 0.14	1.0	0.0386	160 ± 9
Native	0.915	0.917	0.950	2.62 ± 0.06	8.3	0.0163	161 ± 9
A38C-T263C	0.631	0.627	0.630	1.78 ± 0.01	50	0.0154	116 ± 6
5kDa-PEG-conjugate	0.931	0.915	0.911	2.59 ± 0.03	50	0.0123	210 ± 11
Randomly-PEGylated	0.682	0.662	0.702	0.920 ± 0.057	13	0.00738	125 ± 10
Non-conjugated A38C-T263C				0.01	50	0.00010	
Non-conjugated Natural				1.01	13	0.00630	

For the 5kDa-PEG-conjugate and randomly-PEGylated L-asparaginases, the volumetric activity associated to the concentration of non-conjugated L-asparaginase present in the mixture was subtracted from the initial volumetric activity obtained, to later calculate the specific activity. The reported values are average with the standard deviation calculated by error propagation.

[a]One enzymatic unit is 1 μmol of ammonia produced in 10 minutes under the assay conditions.
