# Supplementary material for: Thiol-maleimide poly(ethylene glycol) crosslinking of L-asparaginase subunits at recombinant cysteine residues introduced by mutagenesis
Source: PLoS One. 2018 Jul 27;13(7):e0197643. doi: 10.1371/journal.pone.0197643 (PMC6063399; doi:10.1371/journal.pone.0197643)
Supplement: S5 File — (PDF) [file pone.0197643.s005.pdf]

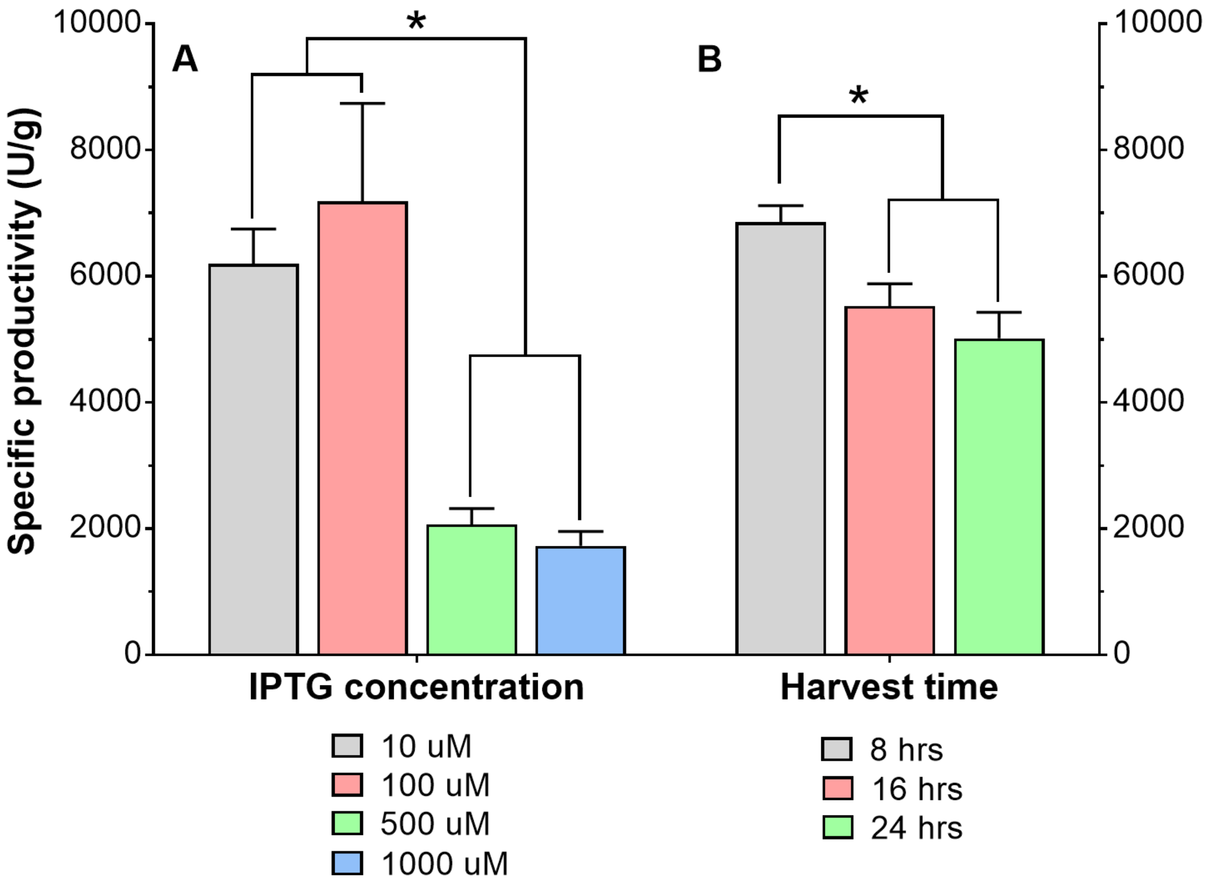

S5 Fig. Optimization of secretory expression. (A) Induction with 10, 100, 500 and 1000 µM IPTG. Cultures were induced at OD_{600nm} 0.200 and grown simultaneously at 37ºC and 220 rpm. Samples were harvested 4 h post-induction. (B) Secretory expression at 8, 16 and 24 h post-induction. Samples were analyzed for asparaginase catalytic activity (U) secreted to the culture medium and normalized by the biomass (g) to express the secretion as specific productivity (U/g). Measurements were performed in triplicate. Each column represents the average with 95% confidence interval. Symbol (*) stands for $P < 0.05$.
