# Supplementary material for: Thiol-maleimide poly(ethylene glycol) crosslinking of L-asparaginase subunits at recombinant cysteine residues introduced by mutagenesis
Source: PLoS One. 2018 Jul 27;13(7):e0197643. doi: 10.1371/journal.pone.0197643 (PMC6063399; doi:10.1371/journal.pone.0197643)
Supplement: S6 File — (PDF) [file pone.0197643.s006.pdf]

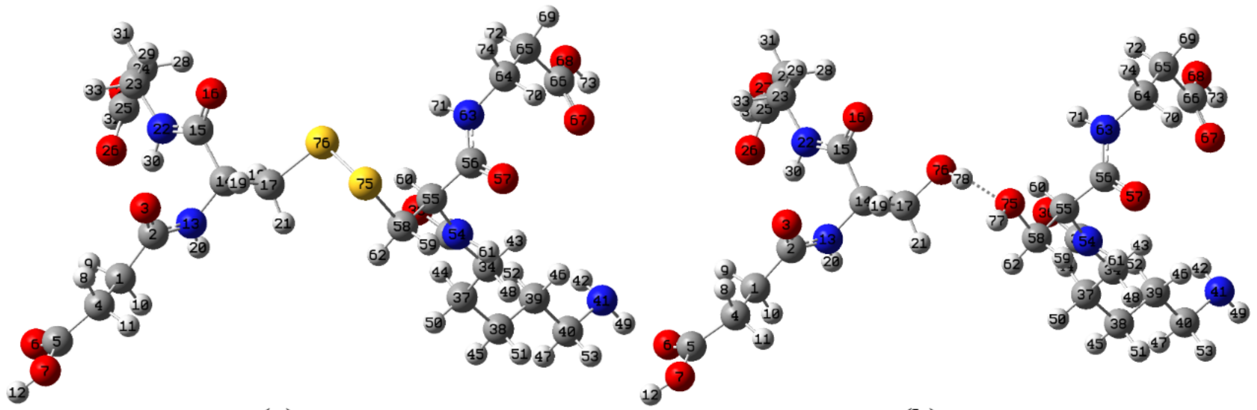

(a) (b)

S6 Fig. Geometrically optimized model structures for C77-105S mutation. Geometrically DFT optimized model structures of L-asparaginase natural Cys-Cys disulfide bond and Ser-Ser mutation. Because of the smaller size of oxygen atoms than sulfur atoms, oxygen atoms (O75 and O76) bound closer to their adjacent carbon atoms (C58 and C17) than sulfur atoms (S75 and S76), which makes longer the distance between two oxygen atoms than two sulfur atoms (O75···O76 = 2.788 Å > S75-S76 = 2.086 Å). However, the distance between oxygen atoms is still close enough to allow the formation of one hydrogen bond (O76H78···O75) between the OH groups. The distance between H78 and O75 is 1.828 Å, which is in the range of a typical hydrogen bond. Also, as our computation, the hydrogen bond energy of O-H···O is 28.9 kJ/mol, showing that it is relatively strong. Except for these changes, there is negligible change in the skeleton of the central structural models, which indicates that the new O-H···O bond is also capable to stabilize this structure like the disulfide bond.
