# Supplementary material for: Thiol-maleimide poly(ethylene glycol) crosslinking of L-asparaginase subunits at recombinant cysteine residues introduced by mutagenesis
Source: PLoS One. 2018 Jul 27;13(7):e0197643. doi: 10.1371/journal.pone.0197643 (PMC6063399; doi:10.1371/journal.pone.0197643)
Supplement: S7 File — (PDF) [file pone.0197643.s007.pdf]

S7 Table. Geometrically optimized bond lengths and angles of model structures.

	Model structure with original S-S bond (X = S)	Model structure with mutated OH···O bond (X = O)
X75···X76	2.086	2.788
O75···H78	-	1.828
O75-H77	-	0.971
O76-H78	-	0.983
C58-X75	1.848	1.435
C17-X76	1.840	1.410
X76···X75-C58	103.79	101.55
C17-X76···X75	103.67	108.27
X75-C58-C55	113.51	107.13
X76-C17-C14	114.69	112.92
