# Supplementary material for: Thiol-maleimide poly(ethylene glycol) crosslinking of L-asparaginase subunits at recombinant cysteine residues introduced by mutagenesis
Source: PLoS One. 2018 Jul 27;13(7):e0197643. doi: 10.1371/journal.pone.0197643 (PMC6063399; doi:10.1371/journal.pone.0197643)
Supplement: S8 File — (PDF) [file pone.0197643.s008.pdf]

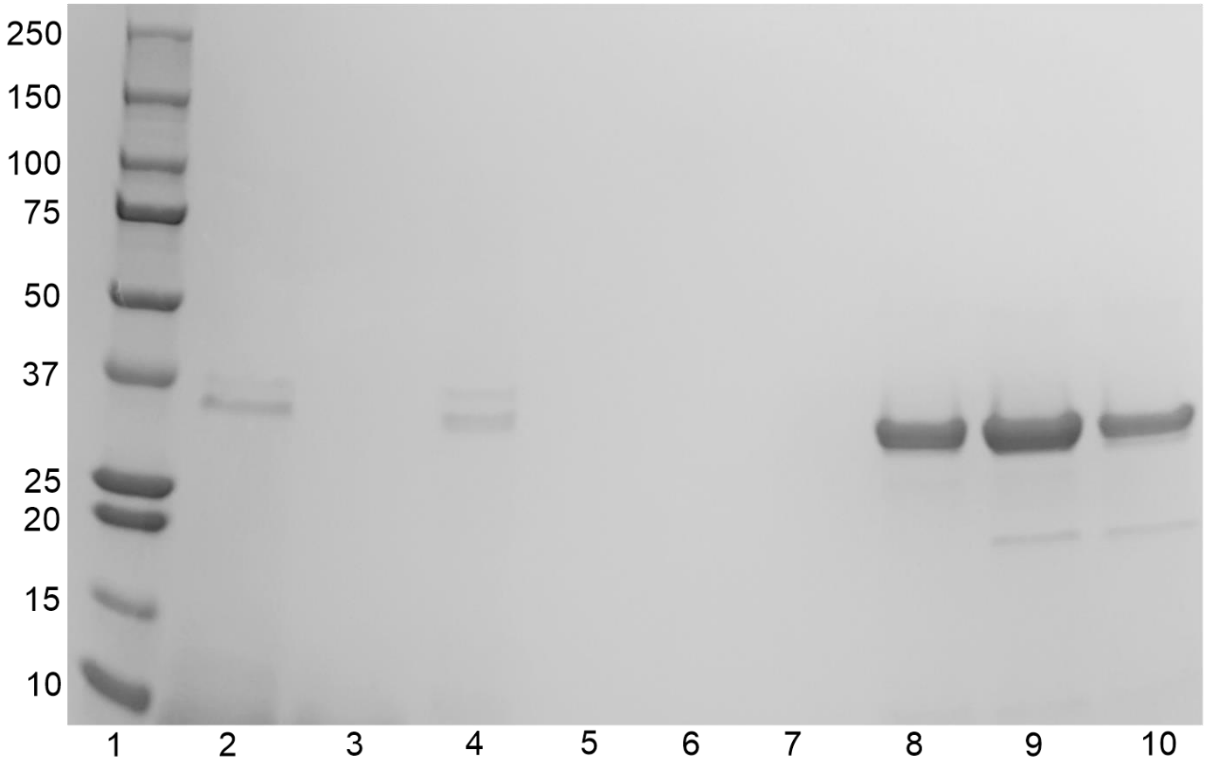

S8 Fig. Purification of recombinant native L-asparaginase. (A) Purification of recombinant native L-asparaginase with a single anion exchange step. [1] Molecular weight marker, [2] crude clear supernatant, [3] ultrafiltration flow-through from 10 kDa cut-off filter, [4] desalted crude from Sephadex G-25, [5] desalting flow-through, [6] load flow-through from the MonoQ column, [7] column wash, [8-10] recombinant native L-asparaginase fractions eluted from the MonoQ (>95% purity).
