# Supplementary material for: Thiol-maleimide poly(ethylene glycol) crosslinking of L-asparaginase subunits at recombinant cysteine residues introduced by mutagenesis
Source: PLoS One. 2018 Jul 27;13(7):e0197643. doi: 10.1371/journal.pone.0197643 (PMC6063399; doi:10.1371/journal.pone.0197643)
Supplement: S9 File — (PDF) [file pone.0197643.s009.pdf]

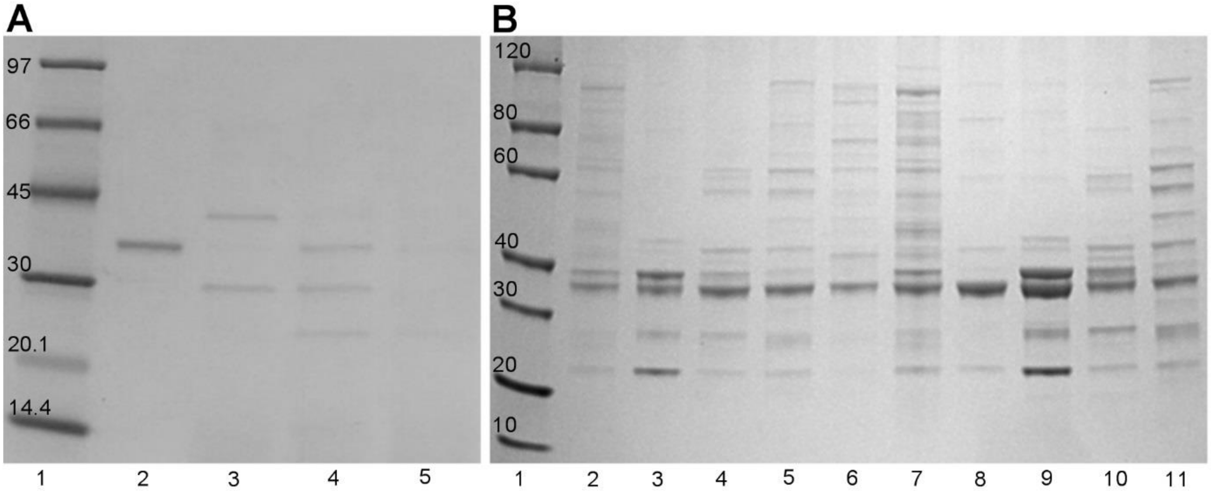

S9 Fig. Purification of L-asparaginase mutants. SDS-PAGE electrophoresis of mutants C77-105S and A38C-T263C. (A) Purification of the mutant C77-105S. [1] Molecular weight marker, [2] commercial natural L-asparaginase control (Millipore Sigma, USA), [3-5] fractions of mutant C77-105S eluted from the MonoQ colunm. (B) Purification of the mutant A38C-T263C. [1] Molecular weight markers, [2] non-reduced crude (no pre-incubation with DTT), [3-6] fractions of mutant A38C-T263C eluted from the MonoQ colunm, [7] reduced crude (pre-incubated with DTT), [8-11] fractions of mutant A38C-T263C eluted from the MonoQ colunm.
