# Supplementary material for: Thiol-maleimide poly(ethylene glycol) crosslinking of L-asparaginase subunits at recombinant cysteine residues introduced by mutagenesis
Source: PLoS One. 2018 Jul 27;13(7):e0197643. doi: 10.1371/journal.pone.0197643 (PMC6063399; doi:10.1371/journal.pone.0197643)
Supplement: S10 File — (PDF) [file pone.0197643.s010.pdf]

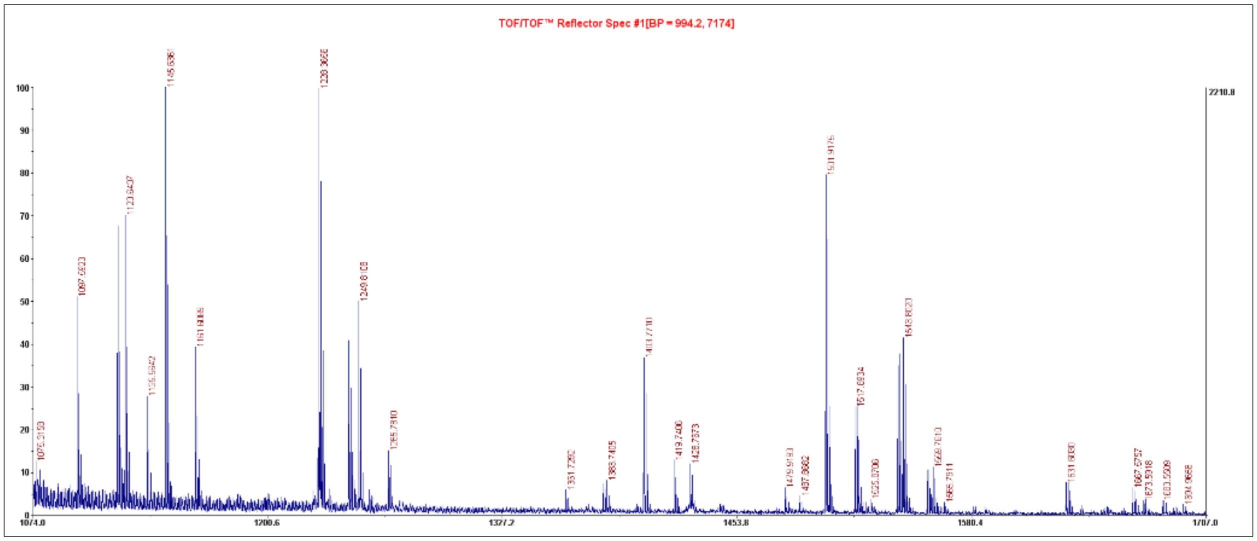

S10 Fig. Precursor peptides pattern of the recombinant native L-asparaginase. *M/z* peaks of trypsin-digested precursors were used to match the recombinant native L-asparaginase against Mascot database. Protein-score was 118, with 46% sequence of *E. coli* L-asparaginase II.
